# Efficacy of minimally invasive tonsil surgery for treatment of obstructive sleep apnea-hypopnea syndrome in children

**DOI:** 10.1590/1414-431X20175846

**Published:** 2017-04-20

**Authors:** X.-Q. Zhang, H. Wang, J. Zhou, P. Zeng, Y. Zhao, Y. Zhang, C. Liu, L.-Q. Jiang, Y.-J. Lan

**Affiliations:** 1 Sleep Medicine Center, West China Fourth Hospital, Sichuan University, Chengdu, Sichuan, China; 2 Department of Occupational Health, West China School of Public Health, Sichuan University, Chengdu, Sichuan, China

**Keywords:** Tonsil, Children, Obstructive sleep apnea-hypopnea syndrome, Polysomnography, Surgery

## Abstract

This study aimed to investigate the efficacy of minimally invasive tonsil surgery for the treatment of obstructive sleep apnea-hypopnea syndrome (OSAHS) in children. Tonsil ablation or turbinate reduction was performed on 49 pediatric patients with OSAHS by minimally invasive tonsil surgery. In order to evaluate the efficacy of surgery, a comparison was conducted between pre-operation and post-operation data in terms of the symptoms, signs and polysomnography test. Total effectiveness rate of the surgery was 83.7%. Subgroup analysis was also performed based on the severity of their conditions: mild, moderate, and severe groups had an effectiveness rate of 90.0, 88.9, and 66.7%, respectively (Hc=6.665, P<0.05). Postoperatively, the apnea-hypopnea index, the minimum oxygen saturation (SaO_2_), and corresponding symptoms improved compared to pre-operation conditions (P<0.05). Minimally invasive tonsil surgery was a safe and effective method for treating OSAHS in children.

## Introduction

Obstructive sleep apnea-hypopnea syndrome (OSAHS) in children refers to a disease in which complete or partial upper airway obstruction occurs frequently during sleep, and consequently disturbs the normal ventilation and sleep structure of children, thus leading to a series of pathophysiologic changes (1,2). OSAHS has severe effects on cognitive function, growth and development, and behavior of children (3). In addition, life quality also significantly decreases (4). The most common causes of OSAHS in children include tonsil hypertrophy, adenoidal hypertrophy, macroglossia, obesity, chronic rhinitis and deviation of nasal septum (5,6). Among these, tonsil and adenoidal hypertrophy are two of the main causes, accounting for over 85% (7). This study shows that immediate surgical treatment can improve life quality and reduce the occurrence of complications in children (8,9).

Tonsillectomy is a common method to treat OSAHS in children. However, traditional tonsillectomy has some disadvantages such as long surgery time and intense intra-operative bleeding. Minimally invasive tonsil surgery is a novel approach used for the treatment of OSAHS in children. This method aims at conducting a minimal invasive ablation on the hypertrophic tonsil, instead of complete resection. The method not only resolves airway obstruction problems, but preserves the immunologic functions of the tonsil. This study evaluated the efficacy of ArthroCare Low-temperature Plasma Surgery System for performing minimally invasive tonsil ablations on 49 pediatric patients with OSAHS.

## Material and Methods

### Patients

This study included 49 pediatric patients diagnosed with OSAHS in the Respiratory and Sleep Disorders Diagnosis and Treatment Center of West China Fourth Hospital from January 2009 to April 2014. Twenty-seven patients were male and 22 were female [average age 8.8±2.09 (4–12 years)]. The most frequent pathology was unilateral or bilateral tonsil hypertrophy grade II (5 patients), grade III (36 patients) and grade IV (8 patients). The main symptoms included snoring accompanied by breath holding and mouth breathing during sleep at night. Part of the patients had a history of chronic rhinitis and adenoidal hypertrophy. However, none had acute tonsil inflammation or combined otitis media. None of the patients had serious congenital malformation, genetic disease, infectious disease, or malignant tumor. The parents of all children provided a signed informed consent.

### Methods

A detailed disease history of the children was conducted. Furthermore, ear-nose-throat examination, adenoid radiography and polysomnography (PSG) were performed in all children. Diagnosis and disease severity classification were conducted according to the Diagnosis and Treatment Guide Draft for Obstructive Sleep Apnea and Hypopnea Syndrome in Children (Urumchi) (5). For example, cases with an apnea-hypopnea index (AHI) >5 and ≤10 are considered mild, >10 and ≤20 are considered moderate and AHI>20 are considered severe. Children were then scheduled for minimally invasive tonsil surgery.

The surgery was carried out under local anesthesia, and the ArthroCare Low-temperature Plasma Surgery System (USA) was adopted to conduct the minimally invasive tonsil ablation. One year after the surgery, the children were re-evaluated with ear-nose-throat examination, adenoid radiography and PSG, and improvement was determined in terms of snoring, breath holding, somnolence and other symptoms. Comparison was performed between pre-operation and post-operation exam results, and levels of signs and symptoms.

### Statistical analysis

The SPSS v21.0 software (IBM, USA) was used for statistical analysis. Quantitative data are reported as means±SD. Gender frequency in each group was compared with the chi-square test. Age and BMI were compared with the single-factor variance analysis for comparison. The paired *t*-test was used for comparisons among the various quantitative data before and after surgery, and for the comparison of ranked data, Kruskal-Wallis test was used. P<0.05 was considered to be statistically significant.

## Results

### General conditions of the subjects

No statistical difference was found in age and gender between mild, moderate, and severe groups. As for BMI, a comparison was performed between any two groups, and the difference among groups was statistically significant, as shown in Table 1.

**Table 1:**
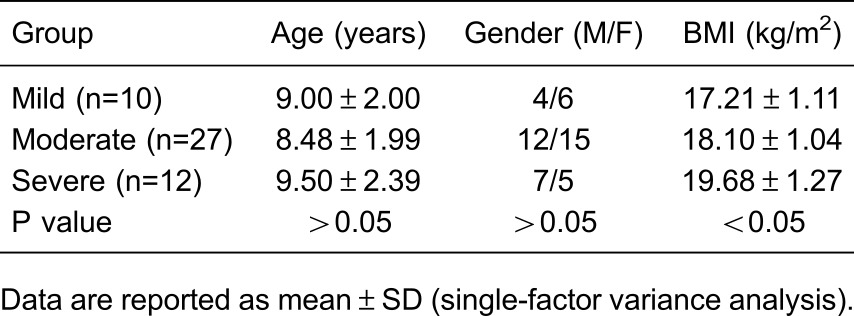
General conditions of the subjects

### PSG main indicators before and after surgery

The AHI and minimum oxygen saturation (SaO_2_) were significantly improved one year after surgery compared with pre-operation values (Table 2).

**Table 2:**
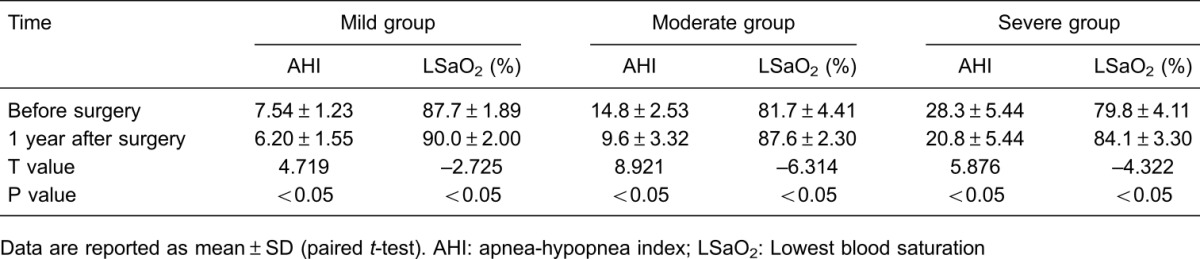
Comparison of polysomnography main indicators before and after surgery

### Improvement of the main symptoms after surgery

Postoperatively, symptoms of snoring, apnea and mouth breathing improved compared with pre-operation symptoms (evaluated as the disappearance of symptoms or their obvious improvement and alleviation). Preoperatively, all 49 patients presented snoring during sleep, of which 45 (91.8%) had improvement; 43 presented apnea, of which 39 (90.7%) had improvement, and 37 presented mouth breathing, of which 34 (91.9%) had improvement (Table 3).

**Table 3:**
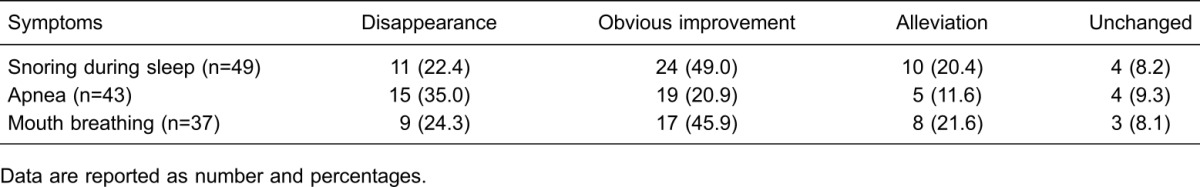
Improvement of symptoms 1 year after surgery

### Surgery efficacy

Effectiveness of the surgery was 83.7% for the 49 patients. Comparison in terms of surgery efficacy between the mild, moderate and severe groups was statistically significant (Hc=6.665, P<0.05). No significant difference was found between mild and moderate groups. However, the severe group had a significantly lower effectiveness rate (Table 4).

**Table 4:**
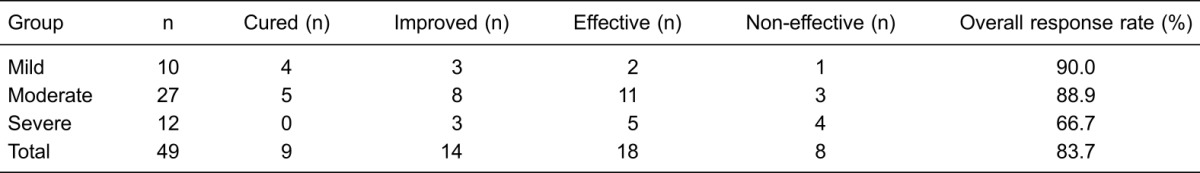
Efficacy of surgery for all patients

## Discussion

OSAHS is a common disease that can occur in children at every stage of growth and development, with the peak period of incidence between 2–6 years of age (10). The incidence rate of OSAHS in children is 1–3%, presenting a rising trend and no difference between genders (11–13). This study confirmed that there was no statistical difference in terms of age and gender proportion for pediatric patients with different disease severities. However, BMI differed in each group. Children with severe conditions had higher BMI, confirming that body weight is a key factor for OSAHS in children (14). Moreover, some studies have shown that the efficacy of surgical treatment in obese children was relatively poorer (15). Therefore, body weight control is a key measure for the prevention and treatment of OSAHS in children.

OSAHS can greatly affect the physical and mental health of children, and has an influence on growth hormone secretion, thus causing growth retardation (16). Long-term oxygen deficit, endocrine disorder and other problems caused by OSAHS may give rise to delayed development of the central nervous system, mental retardation, and cognitive dysfunction. In severe cases, disease of the lungs, heart, brain and other significant organs, as well as behavioral disorders may be induced (17). Therefore, it is very important to treat OSAHS in children.

Minimally invasive tonsil surgery is a new method to treat OSAHS in children. Different from traditional tonsillectomy, this study applied the low-temperature plasma principle to implement the minimally invasive tonsil ablation. An electric active medium at a specific frequency was adopted to generate plasma, and the charged particle decomposed the organized molecular bonds. This causes the directed ablation in the intermolecular site for sectioning, hemostasis, ablation and other functions (18,19). At present, this is the leading technology for treating otorhinolaryngology diseases worldwide (20). This approach does not completely remove the tonsils, which have immunologic functions. It only perforates the tissue to make it smaller, raising concern among scholars due to its novelty. Some studies have shown that tonsillectomy can obviously improve the life quality, cognitive function and clinical symptoms of children patients (21,22).

In our study, 49 pediatric patients with OSAHS underwent minimally invasive tonsil surgery and the improvement rate was above 90%. Obvious contraction was found in the tonsils of these patients. At the same time, obvious expansion was observed in the pharyngeal cavity in our study as reported previously (23). Furthermore, comparison of PSG data before and after surgery showed that the AHI and SaO_2_ improved. Total effectiveness rate of the surgery was 83.7%, which indicates that minimally invasive tonsil surgery can improve snoring and breath-holding in pediatric patients with OSAHS during sleep. However, surgical efficacy was different in children with different severity levels, with worse results in the severe group. The possible cause is that the condition of patients with severe OSAHS was complex and was affected by many factors, such as influences on the nervous and endocrine system due to stenosis of airways, obstruction, obesity, and oxygen deficit.

At present, surgical effectiveness rates of traditional tonsillectomy vary among reports (24–26). This may be related to the severity of the study subjects and the differences in efficacy evaluation indicators. Our study adopted the PSG results to evaluate the efficacy of the surgery, and other factors that lead to snoring in children, including adenoidal hypertrophy and rhinitis. It has been reported that children with partial OSAHS that undergo tonsillectomy have bleeding as the most severe complication after surgery, occurring in 1–20% of cases (27,28). Soon after surgery, such patients can suffer intense pain of the wound, difficulties in eating and drinking, and decreased immunity (29). However, the minimally invasive surgery can not only resolve the symptoms of snoring, but also retain the immunologic function of tonsils in children. Furthermore, this approach can avoid bleeding and pain seen in traditional surgeries. Moreover, adverse reactions are reduced with fewer complications after the surgery (30). Nevertheless, when choosing minimally invasive surgery, attention should be given to indications; if the tonsil is repeatedly infected, it should be removed.

A limitation of this study is the lack of a traditional surgery group. Hence, the comparison between minimally invasive surgery and traditional surgery could not be conducted. Also, adenoidal hypertrophy, rhinitis and other factors were not taken into consideration, which may influence the evaluation of surgery efficacy.

In conclusion, minimally invasive tonsil surgery can effectively improve snoring, apnea, oxygen deficit and other conditions of affected children.
